# The Epithelial-Mesenchymal Transition (EMT) Regulatory Factor SLUG (SNAI2) Is a Downstream Target of SPARC and AKT in Promoting Melanoma Cell Invasion

**DOI:** 10.1371/journal.pone.0040378

**Published:** 2012-07-20

**Authors:** Nina Fenouille, Mélanie Tichet, Maeva Dufies, Anaïs Pottier, Ariane Mogha, Julia K. Soo, Stéphane Rocchi, Aude Mallavialle, Marie-Dominique Galibert, Amir Khammari, Jean-Philippe Lacour, Robert Ballotti, Marcel Deckert, Sophie Tartare-Deckert

**Affiliations:** 1 INSERM, U1065, Centre Méditerranéen de Médecine Moléculaire (C3M), Biologie et Pathologies des Mélanocytes, Nice, France; 2 INSERM, U1065, C3M, Microenvironnement, Signalisation et Cancer, Nice, France; 3 Université de Nice – Sophia Antipolis, Faculté de Médecine, Institut Signalisation et Pathologie (IFR50), Nice, France; 4 CNRS-UMR6290, Université de Rennes1, Centre Hospitalier Universitaire (CHU) Rennes, Rennes, France; 5 Division of Biomedical Sciences, St. George's, University of London, London, United Kingdom; 6 CHU Nantes, Département d’OncoDermatologie, INSERM, U892, Nantes, France; 7 CHU Nice, Hôpital Archet, Service de Dermatologie, Nice, France; University of Birmingham, United Kingdom

## Abstract

During progression of melanoma, malignant melanocytes can be reprogrammed into mesenchymal-like cells through a process similar to epithelial-mesenchymal transition (EMT), which is associated with downregulation of the junctional protein E-cadherin and acquisition of a migratory phenotype. Recent evidence supports a role for SLUG, a transcriptional repressor of E-cadherin, as a melanocyte lineage transcription factor that predisposes to melanoma metastasis. However, the signals responsible for SLUG expression in melanoma are unclear and its role in the invasive phenotype is not fully elucidated. Here, we report that SLUG expression and activation is driven by SPARC (also known as osteonectin), a secreted extracellular matrix-associated factor that promotes EMT-like changes. Ectopic expression or knockdown of SPARC resulted in increased or reduced expression of SLUG, respectively. SLUG increase occurred concomitantly with SPARC-mediated downregulation of E-cadherin and P-cadherin, and induction of mesenchymal traits in human melanocytes and melanoma cells. Pharmacological blockade of PI3 kinase/AKT signaling impeded SPARC-induced SLUG levels and cell migration, whereas adenoviral introduction of constitutively active AKT allowed rescue of SLUG and migratory capabilities of SPARC knockdown cells. We also observed that pharmacological inhibition of oncogenic BRAF^V600E^ using PLX4720 did not influence SLUG expression in melanoma cells harboring BRAF^V600E^. Furthermore, SLUG is a bona fide transcriptional repressor of E-cadherin as well as a regulator of P-cadherin in melanoma cells and its knockdown attenuated invasive behavior and blocked SPARC-enhanced cell migration. Notably, inhibition of cell migration in SPARC-depleted cells was rescued by expression of a SLUG transgene. In freshly isolated metastatic melanoma cells, a positive association between SPARC and SLUG mRNA levels was also found. These findings reveal that autocrine SPARC maintains heightened SLUG expression in melanoma cells and indicate that SPARC may promote EMT-associated tumor invasion by supporting AKT-dependent upregulation of SLUG.

## Introduction

Epithelial to mesenchymal transition (EMT) is a highly conserved developmental program activated during mesoderm formation and neural crest development. This program has also been implicated in promoting dissemination of single malignant cells from primary epithelial tumors [1]. During EMT, cells discard their epithelial characteristics, including cell adhesion and polarity, reorganize their cytoskeleton and acquire a mesenchymal morphology and the ability to migrate. One of the hallmarks of EMT is the functional loss of the cell-cell junction protein E-cadherin. E-cadherin is considered a suppressor of tumor invasion and consistently, loss or partial loss of E-cadherin has been associated with metastatic dissemination and poor prognosis in several solid tumors [1]. Several transcription factors have been identified that can repress E-cadherin expression including SNAIL/SNAI1, SLUG/SNAI2, ZEB1, ZEB2/SIP1, Twist proteins and E47 [2]. These EMT transcription factors bind to E-box elements in the promoter region of E-cadherin leading to transcriptional repression of junctional complexes and induction of the mesenchymal phenotype.

Cutaneous melanoma is an aggressive and potentially fatal form of cancer that derives from melanin-producing melanocytes in the epidermis. Melanocytes originate in the neural crest, a population of highly migratory embryonic cells [3]. Melanoma is a neoplasm of neuroectodermal origin and because of this melanoma cells may not undergo classic EMT-like changes. However, their ability to invade into the dermis is associated with an EMT-like phenotype characterized by changes in expression of cell-cell adhesion molecules of the cadherins family [4], [5]. In normal skin, E-cadherin mediates contacts between melanocyte and adjacent keratinocytes. During melanoma progression, the transition from radial growth phase (RGP) to invasive or vertical growth phase (VGP) is characterized by decreased E-cadherin expression that results in the loss of keratinocyte-mediated growth and motility control [6]. In addition to the loss of E-cadherin, downregulation of other members of classical cadherins such as P- or H-cadherin as well as generation of a truncated secreted form of P-cadherin are frequently observed during progression of melanomas [7]–[9]. Over the past several years, major advances have been made in the identification of genetic factors that contribute to melanoma initiation such as activating mutations in the oncogenes *BRAF* and *NRAS*, however the molecular mechanisms that govern the transition of RGP to VGP with the capacity to metastasize and EMT-related events are still poorly characterized.

SLUG (or SNAI2) belongs to the SNAIL superfamily of zing-finger transcription factors. These developmental proteins are central regulators of EMT during neural crest cell migration and cancer [10]. SLUG is critical for the normal development of neural crest-derived cells [11] and loss-of-function SLUG mutations result in piebaldism and Waardenburg syndrome type 2, two human disorders associated with defective functioning of the embryonic neural crest [12], [13]. SLUG is also required for epithelial cell motility during wound healing and SLUG null mice have retarded epithelial migration rates [14]. More interesting, in melanoma, it was shown that SLUG functions as a melanocyte-specific factor required for the strong metastatic propensity of this tumor [15]. This study also revealed that a gene relevant to embryonic neural crest development might also play a role in the acquisition of the metastatic phenotype. More recently, SLUG was also shown to regulate the transcriptional activity of ZEB1, another important EMT regulator [16]. Apart from its role as an E-cadherin repressor and EMT inducer, SLUG is a survival molecule that promotes resistance to apoptosis in some cell contexts [17]–[20]. In addition, SLUG is a p53 target that antagonizes p53-mediated apoptosis [21]. Whereas SLUG is recognized to drive melanoma metastasis and a key EMT inducer, an important question remains concerning the mechanisms that contribute to the expression and regulation of SLUG during melanoma progression.

Among the proteins whose expression is associated with metastasis and EMT-like changes is the secreted matricellular protein SPARC (also known as osteonectin), which regulates diverse cellular functions, including cell adhesion, migration, cell cycle and survival in a cell-type- and context-dependent manner [22], [23]. Suppression of SPARC expression in human melanoma cells reduced their tumorigenic potential in mouse xenograft assays and inhibited migratory and invasive abilities of human melanoma cells *in vitro* [22], [24], [25]. Conversely, the expression of SPARC in human melanocytes was sufficient to repress E- and P-cadherin and promote epithelial-mesenchymal like transition with acquisition of a migratory phenotype [22]. Our previous study also found that SPARC regulates SNAIL expression in melanocytes and melanoma cells [22]. However, it remains to be addressed whether and how SPARC activates other EMT-inducing transcription factors to suppress E-cadherin and promote cell migration. In this work, we studied further the interplay between SPARC, E- and P-cadherin repression and EMT-like induction in melanoma cells. Our results show SPARC/AKT-dependent regulation of SLUG as an important mechanism underlying EMT-induced cell migration in melanoma.

## Materials and Methods

### Plasmids

The Myc-tagged human SPARC expression plasmid was described previously [22]. Wild-type (−178 wt) and E2 box mutant (mE-pal) mouse E-cadherin promoter constructs fused to luciferase were the generous gifts of Dr. Amparo Cano and have been described previously [26]. Flag-tagged SLUG expression plasmid (Addgene plasmid 25696) was from Dr. Eric R. Fearon.

### Cells and Reagents

Human 501mel cells, kindly supplied by Ruth Halaban were described elsewhere [27]. Human WM9 and WM793 cell lines were the kind gift of Meenhard Herlyn and have been previously described [28]. MeWo and SK-mel28 cell lines were purchased from ATCC. Human melanoma cells were cultured in Dulbecco’s modified Eagle Medium (DMEM) supplemented with 7% fetal bovine serum (Hyclone). Radial growth phase (SGM3 and SGM4) and vertical growth phase, (SGM5 and ME1402) melanoma lines were provided by the Wellcome Trust Functional Genomics Cell Bank at St George’s and described previously [29]. M234, Melan20, M110 and M253b cells were derived from metastatic melanoma tumors by the Dermato-Oncology Department of Nantes University Hospital. A written consent was obtained from the patients for the use of samples and studies were approved by the local ethics committees “Comité de Protection des Personnes Ouest IV-Nantes” and the “Agence Française de Sécurité Sanitaire des Produits de Santé”. Human primary epidermal melanocytes were isolated from foreskin and cultured as described previously [22]. 501mel cells expressing a Myc-tagged SPARC (501mel SPARC clones #1 and #2) or carrying an empty expression cassette of pcDNA3 vector (501mel CTRL) were described earlier [25]. 501mel cells were stably transfected with SLUG expression plasmid and bulk selected in 2 µg/ml puromycin prior to protein analysis and migration assays. Primers and culture reagents were purchased from Invitrogen. AKT inhibitor IV (AIIV) and PI3 kinase inhibitor LY29004 were from Calbiochem. BRAF inhibitor PLX4720 was from Synkinase. Cells were exposed to the various inhibitors at the indicated concentrations for 3 hours. An equal amount of DMSO was used as vehicle control. All other reagents were obtained from Sigma unless stated otherwise.

### Real-time Quantitative PCR

Total RNA was extracted from cell samples using Nucleospin RAII kit (Macherey-Nagel) and following the manufacturer’s instructions. Recovered RNA samples were quantified using NanoDrop spectrophotometer ND1000 (Thermo Fisher Scientific). Reverse transcription was performed on 0.5 µg of total RNA in a volume of 20 µL using High capacity cDNA Reverse Transcription kit (Applied Biosystems) according to the manufacturer’s instructions. Quantitative PCR was performed on 25 ng cDNA samples, in sealed 384-well microtiter plates using the SYBR Green™ PCR Master Mix (Applied Biosystems) with the 7900HT Fast Real-Time PCR System (Applied Biosystems). Transcript relative amounts were determined using the delta-delta-Ct method [30] and human 18S transcript level was used as internal normalizer for each sample. Primer pairs for each cDNA were designed using Primer Express Software (Applied Biosystems). Primer sequences are shown in Table 1.

**Table 1:** Sequences of the primers used in Real-time Q-PCR assays.

Gene product	sense	antisense
SPARC	acatcgggccttgcaaatac	cagtcagaaggttgttgtcctcat
SNAIL	ttctctaggccctggctgc	tacttcttgacatctgagtgggtctg
SLUG	ctgggctggccaaacataag	ccttgtcacagtatttacagctgaaag
TBX2	tcaccatcctaaactccatgc	atgtcgttggctcgcactat
TBX3	gcagctttcaactgcttcg	tgaggttcgatgtccctaca
TWIST1	gcaggacgtgtccagctc	ctggctcttcctcgctgtt
TWIST2	gcaagaagtcgagcgaagat	gctctgcagctcctcgaa
E47	agtacggacgaggtgctgtc	gctttgtccgacttgaggtg
18S	tcggaactgaggccatgatt	cctccgactttcgttcttgatt

### Adenoviral Gene Transduction

Adenoviruses carrying an empty expression cassette of pcDNA3 vector, used as control (AdCTRL) or expressing SPARC with a Myc-tag at its carboxyl terminus were described previously (AdSPARC) [22]. Adenovirus expressing the constitutively active mutant of AKT1, Myr-HA-AKT1 (AdAKT^ca^), was purchased from Vector Biolabs. Adenoviruses were amplified as earlier described [31]. Cells were infected at multiplicity of infection of 5 and assayed 3 days post-infection.

### siRNA Transfection

The control and Stealth® SPARC siRNA duplexes (sequences #1 and #2) were designed by Invitrogen and described previously [25], [32]. Human SLUG and p53 Stealth® siRNAs were purchased from Invitrogen. Transfection of siRNA was carried out using Lipofectamine RNAiMAX (Invitrogen), at a final concentration of 50 nM. Sequences are shown in Table 2.

**Table 2:** siRNAs sequences.

name	sense	antisense
siSPARC#1	AUUUCUUUACAUCAGAAUGGGUCUG	CAGACCCAUUCUGAUGUAAAGAAAU
siSPARC#2	CCACAGUACCGGAUUCUCUCUUUAA	UUAAAGAGAGAAUCCGGUACUGUGG
siSLUG#1	AUUUCUUUACAUCAGAAUGGGUCUG	CAGACCCAUUCUGAUGUAAAGAAAU
siSLUG#2	UUGACCUGUCUGCAAAUGCTT	GCAUUUGCAGACAGGUCAATT
sip53	GCCAAGUCUGUGACUUGCACGUACU	AGUACGUGCAAGUCACAGACUUGGC
siCTRL	CGUACGCGGAAUACUUCGATT	UCGAAGUAUUCCGCGUACGTT

### Cell Lysis and Immunoblotting

Cells were harvested in lysis buffer: 50 mM Tris; 150 mM NaCl; 1% Triton X-100 supplemented with protease inhibitors and phosphatase inhibitors (Roche Diagnostics). Whole cell lysates were subjected to SDS-PAGE and immunoblot analyses done using antibodies to SPARC (Haematologic Technologies), SLUG (Santa Cruz Biotechnology), Myc (Santa Cruz Biotechnology), E-cadherin (BD Biosciences), P-cadherin (BD Biosciences), AKT and phospho-AKT (Ser473) (Cell Signaling Technology), GSK3β (Santa Cruz Biotechnology), phospho-GSK3β (Ser9) (Cell Signaling Technology), phospho-ERK1/2 (Cell Signaling Technology), SNAIL (clone 17EC3, a generous gift of Dr. A. Garcia de Herreros) [33], HSP60 (Santa Cruz Biotechnology), Fibronectin (Sigma) and p53 (Santa Cruz Biotechnology). Peroxidase-conjugated anti-mouse, anti-rabbit and anti-goat antibodies were from Dakopatts. Immunoreactivity was detected with Amersham ECL system.

### Fluorescence and Confocal Microscopy

Cells grown on glass coverslips were rinsed briefly in PBS, fixed in PBS containing 4% formaldehyde, and incubated in a solution containing 0.2% saponin and 1% BSA in PBS for 1 h at room temperature. Cells were incubated with primary antibodies (anti-E-cadherin 1∶100, BD Biosciences; anti-SPARC 1∶50, R&D Systems; anti-SLUG 1∶100, Santa Cruz Biotechnology; anti-Fibronectin 1∶100, BD Biosciences) for 1 h at room temperature. Cells were washed, incubated with Alexa Fluor-conjugated secondary antibodies (1∶1000; Molecular probes), washed again, and mounted in Prolong antifade (Invitrogen). For actin staining, fixed cells were incubated with Texas Red-X phalloidin (1∶100, Molecular Probes) for 1 h at room temperature. Images were captured on a Zeiss LSM 510 META laser scanning confocal microscope, with sequential fluorophore excitation.

### Cell Migration and Three-dimensional (3D) Spheroid Invasion Assay

Chemotaxis assays were monitored using modified Boyden chambers as previously described [25]. Melanoma spheroids were generated using the liquid overlay technique and implanted into a collagen type 1 matrix in growth medium as described [34]. Tumor cell outgrowth was visualized by phase contrast microscopy.

### E-cadherin Promoter Analysis

Transfection of 501mel cells was carried out by using the FuGENE 6 reagent (Roche Applied Sciences). Cells were transfected in 24-well plates with 200 ng of the wild-type (−178 wt) or mutant (mE-pal) luciferase reporter constructs and 50 ng of pCMV-β-galactosidase as a control for transfection efficiency. Cells were harvested 72 hours after transfection and assayed for luciferase activity using the Luciferase assay system (Promega). All experimental values were determined from triplicate wells. Each experiment was repeated at least twice [22], [25].

### Cell-cell Adhesion Assay

The cell aggregation assay was performed essentially as described in [35] with the minor following modifications. Cells were detached by a treatment with HyQTase (Hyclone), plated on bacterial Petri dishes and incubated at 37°C on a gyratory shaker (100 rpm) for 30 min. Clusters of >5 cells were counted from 5 different fields per dish.

### Fluorescent-based Cell Adhesion Assay

The adhesion assays were performed in black 96-well culture dishes in triplicate. Plates were coated overnight at 4°C with 10 µg/mL Fibronectin (BD Biosciences). Cells were collected by HyQTase treatment (Hyclone), labeled with 5 µM CellTracker Green CMFDA (Molecular probes), a cell permeant fluorogenic esterase substrate. After 30 min at 37°C cells were allowed to adhere in serum-free DMEM containing 0.1% fatty-acid-free BSA. At indicated time points, non-adherent cells were removed by washing with PBS supplemented with 1 mM CaCl_2_ and 1 mM MgCl_2_ and fluorescence was measured using a Fluoroskan microplate reader (excitation, 485 nm; emission, 530 nm). Data are expressed as the percentage of adherent cells per total cells.

### Cell Proliferation, Cell Cycle and Apoptosis Analysis

Cell proliferation was determined by a colorimetric assay that is based on the cleavage of yellow tetrazolium salt (XTT) to form an orange formazan dye by mitochondrial dehydrogenases (Cell Proliferation Kit II; Roche Diagnostics). The absorbance of the formazan dye was measured at 490 nm. All experimental values were determined from quadruplicate wells.

Cell cycle profiles were determined by flow cytometric analysis of propidium iodide (PI)-stained cells as previously described [25]. Cells were analyzed using a FACScan (Becton-Dickinson) and the CellQuest software. For flow cytometric analysis of apoptotic death, cells were stained with the Annexin-V-fluos staining kit (Roche Applied Science) according to the manufacturers’ protocol.

### Statistical Analysis

Unless otherwise stated all experiments were repeated at least three times and representative data/images are shown. Error bars indicate ± SD. Student’s *t* and Spearman tests were performed to determine statistical significance. P<0.05 was considered statistically significant.

## Results

### Tumor Cell-derived SPARC Controls SLUG during EMT-like Transition in Melanoma Cells and Melanocytes

**Figure 1:**
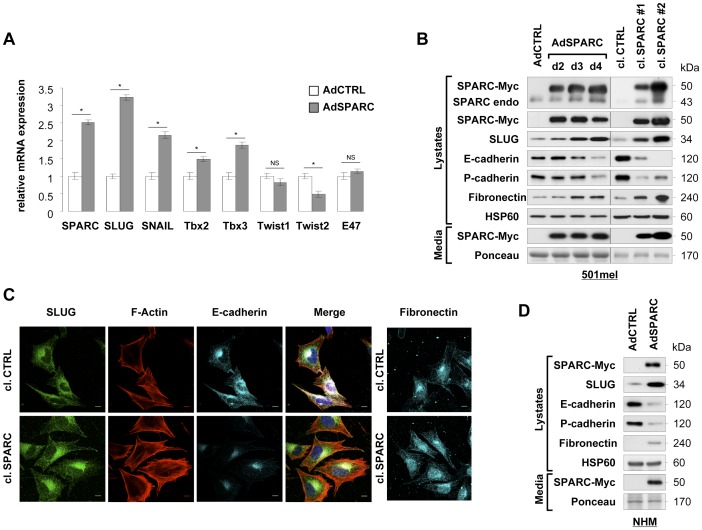
SPARC controls SLUG expression and other markers of the EMT-like transition in melanoma cells and primary melanocytes. (**A**) *Analysis of EMT regulators mRNA levels:* RNAs were prepared from 501mel cells infected with control adenovirus (AdCTRL) or adenovirus-expressing SPARC (AdSPARC) for 3 days. Relative gene expression levels of the indicated E-cadherin repressors were analyzed by SYBR green-based real-time Q-PCR. Data are expressed in arbitrary units as fold change between AdSPARC-infected and control cells. *Columns*, mean of three independent amplifications performed in duplicate; *error bars*, SD. *P<0.05; *NS*, not significant (Student’s test). (**B**) *Expression levels of EMT markers in SPARC overexpressing melanoma cells:* immunoblots of 501mel cells infected with control adenovirus (AdCTRL) or adenovirus-expressing SPARC (AdSPARC) for the indicated times, and of 501mel cell clones expressing SPARC (cl. SPARC #1 and #2) or control vector (cl. CTRL). Total protein lysates and cell supernatants were analyzed for expression of SPARC-Myc transgene, endogenous SPARC, SLUG, E-cadherin, P-cadherin and Fibronectin. HSP60 and Ponceau S-stained bands were used as loading controls in lysates and culture media, respectively. (**C**) *SPARC induces an EMT-like phenotype:* F-actin and immunofluorescent staining of 501mel cell clones expressing SPARC (cl.SPARC) or control vector (cl. CTRL). Cells were co-stained with Texas Red-X phalloidin, anti-SLUG (green) and anti-E-cadherin (cyan) and imaged with confocal microscopy. Merged images of the corresponding three channels are presented. In separate panels, fluorescence signal specific to Fibronectin antibody was visualized as blue. Bars, 10 µm. (**D**) *SPARC promotes EMT-like transition in melanocytes:* immunoblots of normal human melanocytes (NHM) infected with control adenovirus (AdCTRL) or adenovirus-expressing SPARC (AdSPARC) for 4 days. Total protein lysates and cell supernatants were analyzed for expression of the indicated proteins.

We previously showed that SPARC induces E-cadherin repression and EMT-like processes in melanocytes and melanoma cells [22]. To further explore the mechanism of SPARC-mediated E-cadherin silencing, we examined the mRNA expression levels of known E-cadherin repressors that initiate EMT-like transition: SNAIL, SLUG, Twist1, Twist2, E47 [2]. We also examined the levels of Tbx2 and Tbx3, which have been associated with E-cadherin downregulation in melanoma cells [35]. We increased SPARC expression in E-cadherin positive 501mel cells by adenoviral delivery. Real-time Q-PCR analyses showed that overexpression of SPARC in 501mel led to an induction of SLUG, SNAIL, Tbx2 and Tbx3 transcripts (Figure 1A). In contrast, Twist1 and E47 mRNA levels were unaffected by SPARC overexpression. Twist2 mRNA, however, was slightly decreased in cells transduced by AdSPARC compared to control cells. SNAIL mRNA induction following SPARC expression was consistent with our previous observations [22] that SPARC induced SNAIL in human primary melanocytes. At protein level, we confirmed upregulation of SLUG and SNAIL by adenoviral-delivered SPARC expression (Figure 1B, left; Figure S1A), whereas levels of Tbx2 and Tbx3 were unaffected (Figure S1A). Interestingly, SLUG protein induction followed the expression of SPARC-Myc transgene in the culture media, and was temporally correlated with decreased levels of E-cadherin and P-cadherin and increased expression of the mesenchymal marker Fibronectin (Figure 1B, left). Induction of SLUG mRNA and protein levels as well as alterations of EMT-associated proteins upon SPARC overexpression were also observed in clones of 501mel stably transfected with SPARC (Figure 1B, right; Figure S1C). These EMT-like morphological changes induced by SPARC were confirmed by immunofluorescent staining of SLUG, E-cadherin and Fibronectin. In addition, F-actin staining revealed more stress fibers in SPARC overexpressing cells than in control cells (Figure 1C).

We next asked whether SLUG was regulated by SPARC in normal human melanocytes (NHM). Primary melanocytes were infected with an adenovirus control or expressing SPARC for 4 days and expression of SLUG was analyzed by immunoblotting (Figure 1D). We found that melanocytes modified to produce and secrete SPARC showed a marked increase in SLUG expression compared with control cells. Upregulation of SLUG was associated with loss of E-cadherin and P-cadherin expression and with a significant increase in Fibronectin. The latter finding was confirmed by densitometric analyses of the immunoblots (Figure S1B).

Collectively, these data indicate that SPARC-induced EMT-like transition in melanocytes and melanoma cells is accompanied by upregulation of SLUG, and suggest that SPARC functions by maintaining the augmented expression of SLUG in melanomas.

**Figure 2:**
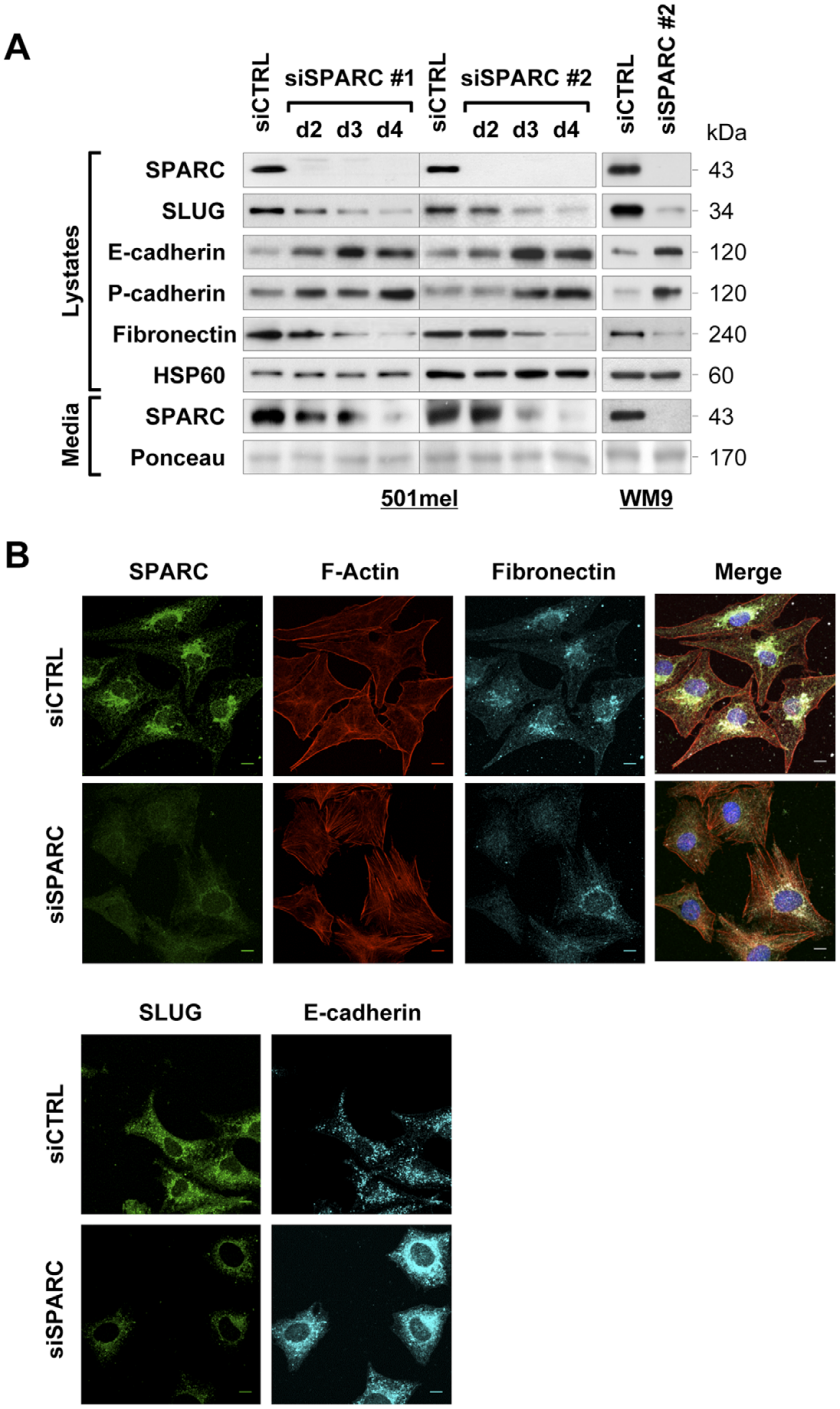
siRNA-mediated knockdown of SPARC decreases SLUG and restores E-cadherin and P-cadherin expression. (**A**) *Expression levels of EMT markers in SPARC-depleted cells:* immunoblots of 501mel cells transfected with control siRNA (siCTRL) for 4 days or two SPARC siRNAs for the indicated times (left), and of WM9 cells transfected with siCTRL or siSPARC #2 for 4 days (right). Total protein lysates and cell supernatants were analyzed for expression of the indicated proteins. (**B**) *Morphology of SPARC-depleted cells: F*-actin and immunofluorescent staining of 501mel cells transfected with control siRNA (siCTRL) or SPARC siRNA (siSPARC). After 4 days, cells were co-stained with Texas Red-X phalloidin, anti-SPARC (green) and anti-Fibronectin (cyan) and imaged with confocal microscopy. Merged images of the corresponding three channels are presented. *Bottom panels,* expression of SLUG (green) and E-cadherin (cyan) was analyzed by immunofluorescence staining. Bars, 10 µm.

We next examined whether silencing of SPARC in melanoma cells could affect SLUG protein levels. As we recently showed that siRNA-mediated depletion of SPARC after a 5-day period promotes spontaneous apoptosis [36], we investigated the effect of SPARC depletion in a suitable time window, in which SPARC knockdown did not induce cell death (Figure S1D). To diminish the likelihood of RNAi off-targets effects, we used two independent non-overlapping siRNAs to silence SPARC in 501mel cells. We found that reduction of SPARC in culture media was concomitant with a time-dependent decrease in SLUG and Fibronectin expression and increase in E- and P-cadherins protein levels (Figure 2A, left). A similar robust decrease of SLUG expression upon SPARC knockdown was observed in WM9 melanoma cells (Figure 2A, right). Immunofluorescence microscopy confirmed decreased levels of SLUG and Fibronectin and increased levels of E-cadherin in SPARC knockdown cells compared with control cells (Figure 2B), suggesting that SPARC knockdown cells reverses the EMT-like phenotype. However, F-actin staining revealed no significant differences in cell morphology between control and siSPARC-transfected cells, but an increase in stress fibers formation in SPARC knockdown cells.

### The Regulation of SLUG Protein Levels by SPARC is Independent of p53

**Figure 3:**
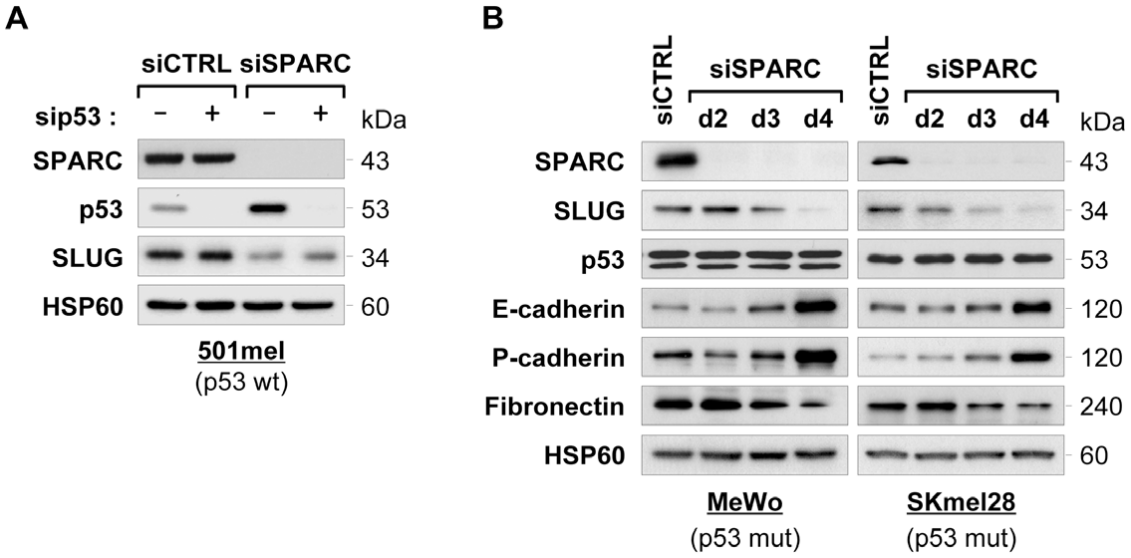
siRNA-mediated knockdown of SPARC decreases SLUG expression in a p53-independent manner. (**A**) *Analysis of SLUG expression in p53 and SPARC co-depleted cells:* 501mel cells were transfected for 3 days with control siRNA (siCTRL), p53 siRNA (sip53), SPARC siRNA (siSPARC) alone or in combination at 50 nM final concentration. Total protein lysates from resulting cells were analyzed by immunoblotting for expression of SPARC, p53, SLUG and HSP60 (loading control), as indicated. (**B**) *Analysis of SLUG expression after SPARC depletion in p53-mutated melanoma cells:* MeWo and SKmel28 cells were transfected with control siCTRL for 4 days or siSPARC for the indicated times. Expression levels of SPARC, SLUG, p53 E-cadherin, P-cadherin, Fibronectin and HSP60 (loading control) were analyzed by immunoblotting.

We recently demonstrated that SPARC suppresses p53 functions in melanoma cells. Notably, we found that p53 is stabilized and activated upon RNAi-mediated knockdown of SPARC [36]. As p53 was shown to induce MDM2-mediated SLUG degradation in lung cancer cells [37], we asked whether downregulation of SLUG was mediated through p53 in SPARC knockdown cells. We used siRNA to target p53 in wild-type p53 501mel cells and melanoma cells containing mutant copies of p53. Treatment with p53 siRNA led to an abolition of p53 in 501mel cells and prevented SPARC siRNA-mediated accumulation of p53 (Figure 3A). Blocking p53 activation in SPARC-depleted cells had no effect on the reduction of SLUG protein levels mediated by SPARC knockdown. Accordingly, in p53-mutated MeWo and SKmel28 cells, we still observed the downregulation of SLUG upon siRNA-mediated SPARC knockdown and the regained expression of E- and P-cadherin along with reduction of Fibronectin levels (Figure 3B). These findings indicate that the effects of SPARC knockdown on suppression of SLUG and reversion of the EMT-like process did not require activation of the p53 pathway.

### Role of PI3 Kinase and AKT in SPARC-induced SLUG Expression and Melanoma Cell Migration and Invasion

**Figure 4:**
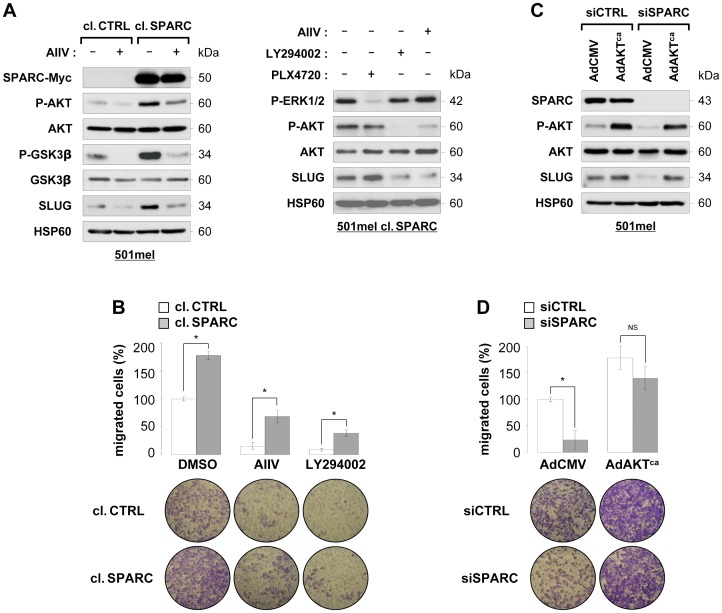
SPARC-mediated SLUG expression and promotion of melanoma cell migration is PI3K/AKT dependent. (**A**) *Influence of PI3K/AKT and BRAFV600E signaling on SLUG expression:* left panel, 501mel cells control (cl. CTRL) or overexpressing SPARC (cl. SPARC) were treated with DMSO or 10 mmol/L AKT inhibitor IV (AIIV). Immunoblots show the phosphorylation status of AKT (Ser473) and GSK3β (Ser9) and expression levels of SPARC-Myc transgene, AKT, GSK3β and SLUG. *Right panel*, 501mel cl. SPARC cells were treated with DMSO or 10 mmol/L AIIV, LY294002 or PLX4720 for 3 hours. Immunoblots show the phosphorylation status of MAP kinases ERK and AKT and expression levels of SLUG. HSP60 was used as a loading control. (**B**) *Influence of PI3K/AKT signaling on cell migration:* serum-stimulated cell migration was analyzed using Boyden chamber assays in control and SPARC 501mel cells treated with DMSO or 10 mmol/L AIIV or LY294002 as indicated. Results are expressed in percent of control. *Columns*, means of triplicates from two independent experiments; *error bars*, SD. *P<0.05 (Student’s test). Representative images of lower surface of membranes are shown. (**C**) *Expression of Myr-AKT protects from SLUG decrease after SPARC depletion:* 501mel cells were infected with a control empty adenovirus (AdCMV) or with adenovirus encoding the constitutively active mutant Myr-HA-AKT1 (AdAKT^ca^) and transfected 6 hours later with 50 nM siCTRL or siSPARC for 3 days. Cells were harvested and proteins were analyzed by immunolotting using the indicated antibodies. (**D**) *Expression of Myr-AKT rescues migratory defects of SPARC-depleted cells:* serum-stimulated cell migration was analyzed using Boyden chamber assays in 501mel cells infected with AdCMV or AdAKT^ca^ and after depletion for endogenous SPARC as described above. Results are expressed in percent of control. *Columns*, means of triplicates from two independent experiments; *error bars*, SD. P<0.05; *NS*, not significant (Student’s test). Representative images of lower surface of membranes are shown.

We sought to identify the signaling pathways mediating SPARC-induced SLUG expression. The recent observation that SPARC stimulates PI3 kinase/AKT-dependent pathway in A375 melanoma cells [36] prompted us to examine the role of this pathway in SPARC-driven SLUG expression and promotion of melanoma cell migration. We first examined the effect of pharmacological inhibition of AKT on SLUG expression in control and SPARC-overexpressing 501mel cells. As shown in Figure 4A, SPARC overexpression was associated with increased levels of AKT phosphorylated on Ser473. Addition of AKT inhibitor IV (AIIV) efficiently blocked phosphorylation of AKT and of its downstream target GSK3β on Ser9 and lowered levels of SLUG in both control and SPARC overexpressing cells. Consistently, pharmacologic blockade of PI3 kinase activity by LY294002 also reduced SLUG protein levels in 501mel SPARC cells. Like the majority of melanoma tumors, 501mel cells harbor the oncogenic BRAF^V600E^mutation and consequently constitutive activation of the MAP kinase/ERK signaling pathway. We thus examined the effect of a specific BRAF inhibitor (PLX4720) [38] on SLUG protein levels. Treatment with PLX4720 for 3 hours inhibited constitutive MAP kinase/ERK phosphorylation but had no major effect on SLUG protein levels (Figure 4A, right pannel). Similar observations were made after prolonged exposure to PLX4720 (data not shown). This suggests that oncogenic BRAF signaling does not decrease SLUG expression in BRAF^V600E^ melanoma cells. In migration assays, pharmacologic blockade of PI3 kinase and AKT by LY294002 and AIIV, respectively inhibited basal levels of cell migration and blocked SPARC-enhanced migration (Figure 4B).

To further assess the contribution of AKT, we tested whether a constitutively active form of AKT can rescue SLUG and the migratory phenotype of SPARC knockdown cells. 501mel cells were infected with an adenovirus control (AdCMV) or expressing Myr-AKT (AdAKT^ca^) and then transfected with control or SPARC siRNA. SPARC knockdown significantly reduced phosphorylation of AKT and SLUG expression (Figure 4C). Forced expression of constitutively active AKT into 501mel cells resulted in its constitutive phosphorylation and increased cell migration (Figure 4C and D). Interestingly, adenoviral delivery of constitutively active AKT allowed rescue of SLUG expression and migratory capabilities of SPARC knockdown cells. Thus, SPARC-mediated SLUG expression and promotion of cell migration requires AKT activation. These data also reveal a potential link between PI3 Kinase/AKT-regulated cell migration and SLUG expression in melanomas.

**Figure 5:**
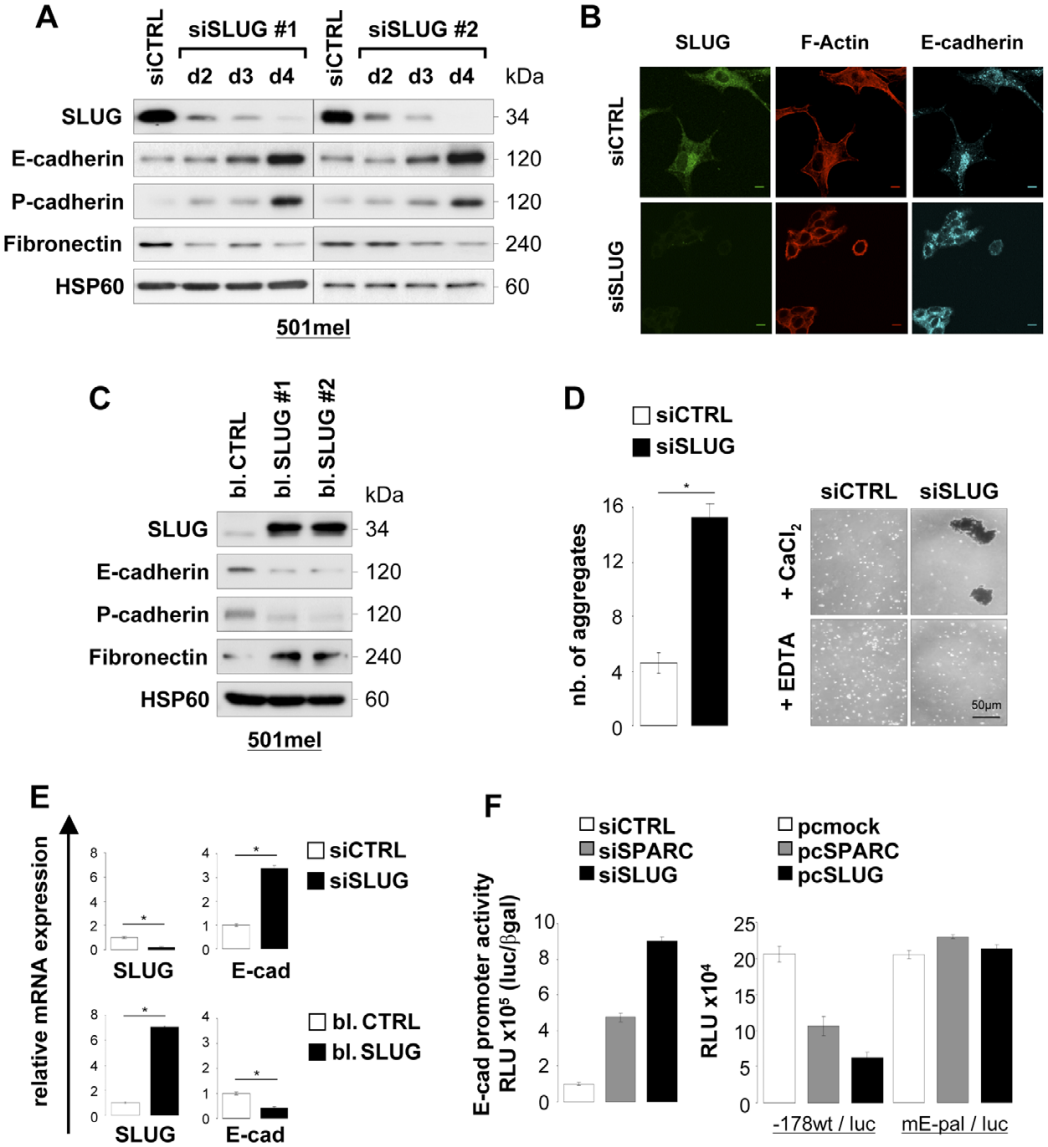
Knockdown or overexpression of SLUG modulates E-cadherin and P-cadherin adhesion molecules. (**A**) *Expression levels of EMT markers in SLUG-depleted cells:* 501mel cells were transfected with control siRNA (siCTRL) for 4 days or two SLUG siRNAs (siSLUG #1 and #2) for the indicated times. Expression levels of SLUG, E-cadherin, P-cadherin and Fibronectin were analyzed by immunoblotting. HSP60 was used as loading control. (**B**) *Morphology of SLUG-depleted cells:* expression of SLUG (green), E-cadherin (cyan) and actin cytoskeleton (Texas Red-X phalloidin) following siRNA-mediated SLUG depletion in 501mel cells was analyzed by fluorescence staining and confocal microscopy. Bars, 10 µm. (**C**) *Ectopic SLUG expression induces an EMT-like phenotype:* control (bl. CTRL) or SLUG-overexpressing (bl. SLUG #1 and #2) 501mel cell populations were analyzed by immunoblotting for expression of SLUG, E-cadherin, P-cadherin, Fibronectin and HSP60 (loading control). (**D**) *Depletion of SLUG increases Ca^2+^-dependent cell-cell adhesion:* adhesion assays were performed as described in the Materials and Methods after treatment of 501mel cells with siCTRL or siSLUG as indicated. The phase-contrast pictures show aggregates formed in presence of 1 mmol/L CaCl_2_ alone or with 3 mmol/L EDTA. The average of two independent adhesion assays and SD are presented. *Columns*, average of two independent assays; *error bars*, SD. *P<0.05 (Student’s test). (**E**) *SLUG regulates E-cadherin mRNA levels:* RNAs were prepared from 501mel cells transfected with siCTRL or siSLUG for 4 days, and from bulk selected control or SLUG-overexpressing 501mel cells. The relative mRNA expression levels of SLUG and E-cadherin were measured by SYBR green-based real-time Q-PCR. *Columns*, mean of three independent amplifications performed in duplicate; *error bars*, SD. *P<0.05 (Student’s test). (**F**) *E-cadherin promoter activity:* 501mel cells were transfected with siCTRL, siSPARC or siSLUG, and 24 hours later with wild-type E-cadherin promoter reporter construct (left). 501mel cells were co-transfected with an empty vector (mock) or vectors expressing SPARC or SLUG and wild-type (−178 wt/luc) or mutant (mE-pal/luc) E-cadherin promoter reporter constructs (right). After 3 days, luciferase activities were measured and normalized to β-galactosidase activities. *Columns*, mean of triplicates; *errors bars*, SD.

To assess the role of SLUG in the control of the cell-cell adhesion molecules E- and P-cadherins in melanoma cells, we carried out knockdown experiments using two differents non-overlapping siRNAs specifically targeting SLUG, and gain-of-function experiments where 501mel cells were stably transfected with SLUG. As shown in Figure 5A, knockdown of SLUG led to regained expression of both E- and P-cadherins in a time-dependent manner. A slight decrease in Fibronectin protein expression was also observed in SLUG silenced 501mel cells compared to control siRNA-treated cells. Increased expression of E-cadherin in SLUG knockdown cells was also confirmed by immunofluorescence analysis (Figure 5B). In addition, SLUG-deficient cells adopted a rounded morphology with an altered actin cytoskeleton and a loss of the characteristic fibroblastic-like morphology, as revealed by fluorescent F-actin staining (Figure 5B). In SLUG-overexpressing 501mel cell populations, we found reduced E- and P-cadherins protein levels and increased Fibronectin expression (Figure 5C). We next tested if SLUG depletion would have a functional impact on Ca^2+^-dependent cell-cell adherence. 501mel cells were transfected with control or SLUG siRNA and an *in vitro* cell-cell adhesion assay was performed in presence or absence of Ca^2+^ ions (+ EDTA). The results shown in Figure 5D revealed an increase in cell aggregates from SLUG-depleted cells compared to control cells, and that formation of cell aggregates was prevented upon treatment with the chelating agent EDTA. Thus, knockdown of SLUG increases Ca^2+^-dependent cell-cell adhesion. In the next series of experiments, we confirmed that SLUG is a transcriptional repressor of E-cadherin in 501mel cells [26], [39]. Analysis of E-cadherin mRNA levels by real-time Q-PCR upon SLUG depletion or ectopic expression showed an increase or decrease of E-cadherin transcript, respectively (Figure 5E). We also examined the effect of SLUG on the activity of the mouse E-cadherin promoter in 501mel cells (Figure 5F). Luciferase reporter assays show that depletion of SPARC or SLUG by siRNA increased the activity of the E-cadherin promoter and conversely, expression of SPARC or SLUG repressed promoter activity. No repression was seen when the E-cadherin promoter is mutated in the E-box elements (mE-pal construct). Similar responses to SLUG knockdown or SLUG ectopic expression were seen in other melanoma cell lines (Figure S2).

Because siRNA SPARC-induced migratory inhibition coincides with SLUG reduction, we next analyzed the effect of SLUG knockdown on melanoma cell migration and invasive behavior. Knockdown of SPARC or SLUG with siRNAs in several melanoma cells strongly inhibited melanoma cell migration in Boyden chambers assays, compared with control cells (Figure S3). These data confirm previous observations and indicate that SPARC and SLUG are both important regulators of melanoma cell migration.

Although SLUG is recognized as a pro-survival factor in some cell types and melanocytes, some studies showed that its depletion in melanoma cells was not associated with significant changes in proliferation and survival [15], [20]. Similarly, we found that siRNA-mediated depletion of SLUG did not alter cell proliferation and cell cycle profiles, and was not associated with significant apoptosis over a 4-day period (Figure S4). Therefore, apoptosis does not account for the inhibitory effects on cell migration observed in SLUG knockdown cells.

**Figure 6:**
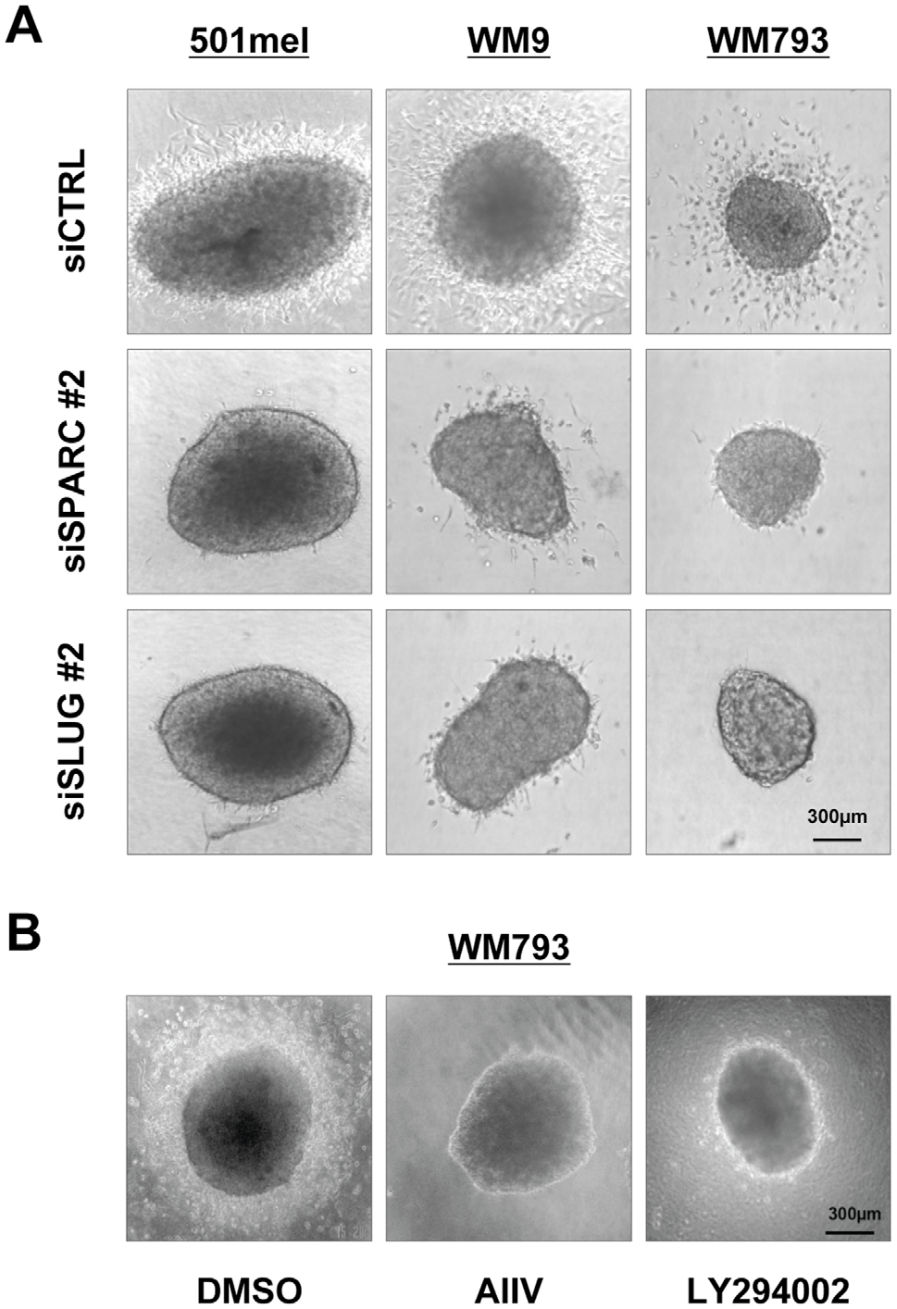
siRNA-mediated knockdown of SPARC or SLUG and inhibition of AKT signaling reduce invasion into 3-dimensional collagen matrix. (**A**) *Depletion of SPARC or SLUG decreases tumor spheroid invasion:* preformed melanoma spheroids of 501mel, WM9 and WM793 cells transfected with siCTRL, siSPARC or siSLUG as indicated were implanted into a gel of collagen type I. Spheroids were incubated in growth medium for 3 days and tumor cell outgrowth was visualized by phase contrast microscopy. (**B**) *Influence of PI3K/AKT signaling on spheroid invasion:* preformed melanoma spheroids of 501mel, WM9 and WM793 were implanted into a gel of collagen type I and incubated in growth medium for 3 days with with DMSO or 10 mmol/L AIIV or LY294002. Tumor cell invasion was visualized as above. Representative example of spheroids from each culture is shown.

Tumor migration was then analyzed in a more physiological context; 501mel, WM9 or WM793 cells were grown as spheroids embedded in collagen, an assay that mimics 3-dimensional growth and invasion by melanoma cells [40]. Knockdown of SPARC or SLUG had no effect on spheroid growth rate but significantly reduced cell invasion into collagen (Figure 6A). Thus, SPARC and SLUG expression is required for tumor invasion in 3-dimensional cultures. Consistent with the regulation of SLUG by the PI3 kinase/AKT pathway (see Figure 4), pharmacologic inhibition of this signaling pathway by LY294002 or AIIV inhibited the invasive phenotype of melanoma spheroids into collagen (Figure 6B).

It has remained unclear how SLUG affects cell migration. We observed that Slug-deficient cells showed altered actin cytoskeletal organization (Figure 5B). To determine if the migration defect of SLUG-depleted melanoma cells was related to an alteration in cell adhesion, a fluorescent adhesion assay was performed with Fibronectin. Suppression of SLUG led to decreased attachment of 501mel, MeWo and SKmel28 cells on Fibronectin (Figure S5). Thus, some migratory defects of SLUG knockdown cells may occur through alterations in cell-substratum interactions and cytoskeleton reorganization.

### SLUG is Required for SPARC-mediated Melanoma Cell Migration

**Figure 7:**
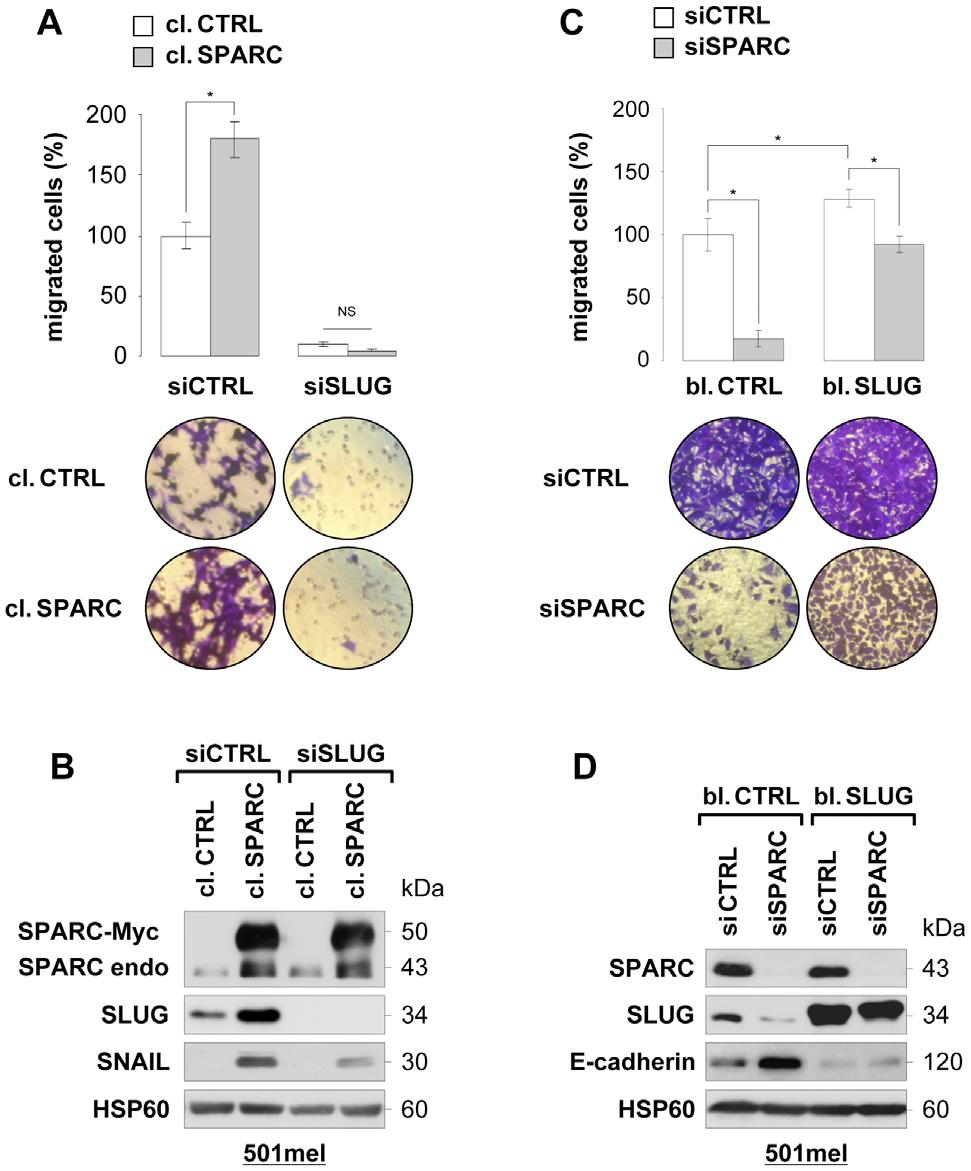
SLUG plays a critical role in SPARC-mediated melanoma cell migration. (**A**) *Depletion of SLUG blunts SPARC-induced cell migration:* 501mel cells control (cl. CTRL) or overexpressing SPARC (cl. SPARC) were transfected with control siRNA (siCTRL) or SLUG siRNAs (siSLUG) for 4 days. Chemotaxis was assassed using Boyden chamber assays. Cells were left to migrate for 20 hours, then fixed, stained and counted. Results are expressed in percent of control. *Columns*, means of triplicates from two independent experiments; *error bars*, SD. *P<0.05; *NS*, not significant (Student’s test). Representative images of lower surface of membranes are shown. (**B**) *Analysis of SLUG expression:* levels of SPARC-Myc transgene, endogenous SPARC, SLUG, SNAIL and HSP60 (loading control) in the resulting cells were analyzed by immunoblotting. (**C**) *Ectopic expression of SLUG bypasses migratory defects of SPARC-depleted cells:* Control (bl. CTRL) or SLUG-overexpressing (bl. SLUG) 501mel cells were transfected with control siRNA (siCTRL) or SPARC siRNAs (siSPARC) for 4 days. Chemotaxis was assessed using Boyden chamber assays as described in (A). *P<0.05 (Student’s test). (**D**) *Analysis of E-cadherin expression:* levels of SPARC, SLUG, E-cadherin and HSP60 (loading control) in the resulting cells were analyzed by immunoblotting.

To show that SLUG is a downstream target of SPARC in melanoma cells, we knocked down SLUG in SPARC-overexpressing 501mel cells. As shown in Figure 7A, knockdown of SLUG abrogated the effect of SPARC-enhanced cell migration as well as basal migration of 501mel cells. Immunoblot analysis shows the efficient depletion of SLUG in control cells and cells stably transfected with SPARC (Figure 7B). As a control, we also analyzed SNAIL expression and found its upregulation in SPARC-overexpressing cells, which is consistent with our early study showing that SPARC increased SNAIL expression in human primary melanocytes [22].

To provide further functional link between SPARC and SLUG in controlling EMT-induced cell migration, we next examined if ectopic expression of SLUG was able to protect from the migratory phenotype of SPARC knockdown cells. Control 501mel cells or stably transfected with SLUG were treated with SPARC siRNA and cell migration assays were performed 4 days later. Ectopic expression of SLUG was able to protect from the knockdown phenotype on migration (Figure 7C) and reinduction of E-cadherin (Figure 7D). Figure 7D also shows that SPARC level was dramatically reduced following siRNA treatment, and that endogenous SLUG level was reduced upon SPARC depletion. Thus, SLUG functionally contributes to SPARC-enhanced melanoma cell migration and E-cadherin repression.

### Relationship between SPARC and SLUG Levels in Melanoma Cells at Various Stages of Tumor Development

**Figure 8:**
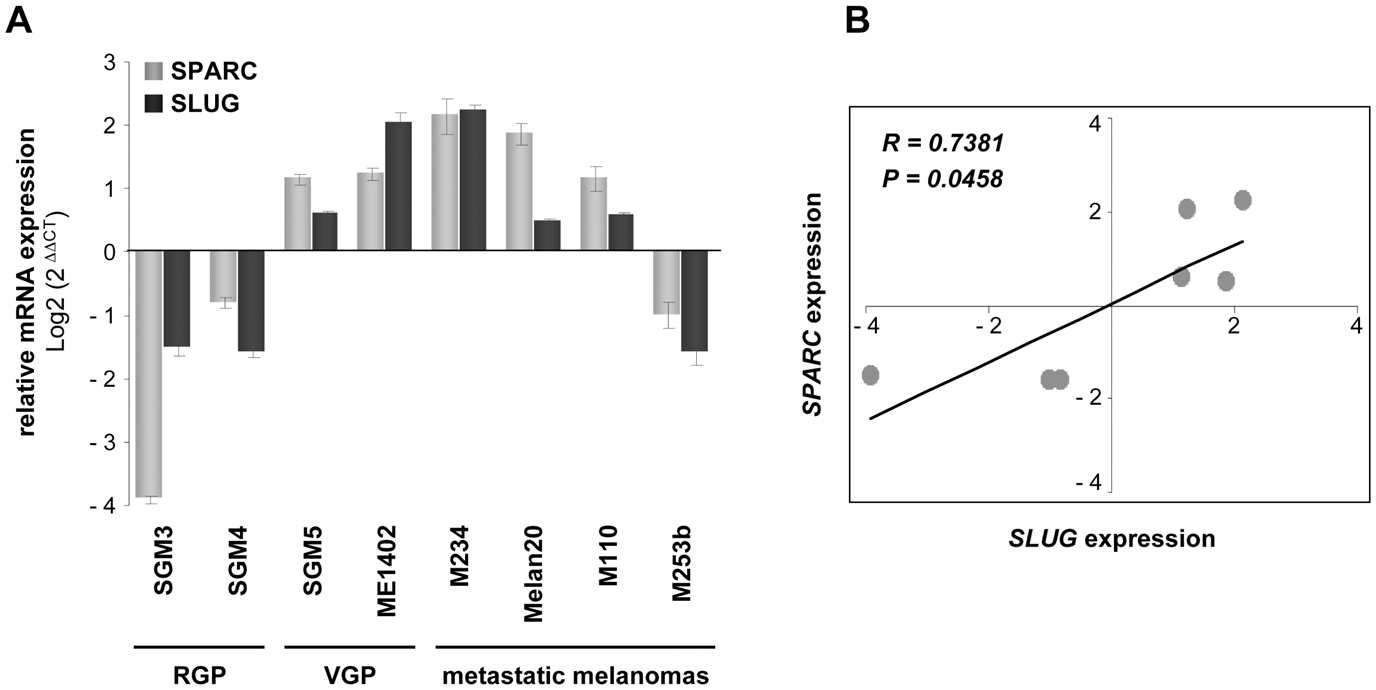
mRNA levels of SPARC and SLUG in melanoma cells at various stages of tumor development. (**A**) *Analysis of SPARC and SLUG mRNA levels:* relative gene expression levels of SPARC and SLUG in cultures of melanoma cells derived from RGP, VGP or metastatic melanoma tumors were evaluated by relative Q-PCR using an ABI Biosystems 7900HT Fast Real Time PCR System and the SYBR Green dye detection protocol. Data were analyzed using the 2^−ddCt^ method and human 18S transcript level was used to normalize for each sample. Values are the mean of independent triplicates. RGP, Radial Growth Phase; VGP, Vertical Growth Phase. (**B**) *Positive correlation between SPARC and SLUG in melanoma samples:* regression analysis to determine the correlation between *SPARC* and *SLUG* in human melanoma samples. *R*, Spearman’s Rank Correlation Coefficient; P<0.05 (Spearman’s test).

Finally, we have examined the expression profiles of SPARC and SLUG in a small panel of cultures of melanoma cells derived from RGP or VGP primary tumors or from lymph node melanoma metastases (Figure 8). Real-time Q-PCR analysis revealed a positive association between SPARC and SLUG transcripts in freshly isolated melanoma samples tested. The positive expression of SPARC mRNA observed in the two VGP melanoma samples confirmed our previous observations that SPARC is induced during the RGP to VGP transition of melanomas [22]. The correlation (*R*) and *P* values between SLUG and SPARC levels were determined by regression analysis. These data provide independent support for a potential functional link between SPARC and SLUG during melanoma progression.

## Discussion

SPARC is widely expressed in cancer and is thought to promote tumor progression through regulation of cell survival, invasiveness and tumor-host interactions [23], [41]. Several studies from our laboratory and others have shown that inhibition of SPARC in both malignant cells *in vitro* [22], [24] and tumors *in vivo* [24], [25], [42] reduces melanoma growth, invasiveness, and induces spontaneous apoptotic cell death and anti-tumor cytotoxic capacity. One mechanism underlying SPARC function in melanoma appears to be E-cadherin repression and promotion of an EMT-like transition [22], [43], a process having a central role during RGP to VGP progression. In this study, we show that SPARC regulates the EMT regulatory factor SLUG and demonstrate the critical role of the PI3 kinase/AKT pathway in this process.

Much evidence exists to support an important role for the protein kinase B/AKT in melanomagenesis. Activated AKT was found in 70% of human melanomas [44]. Several mechanisms may contribute to this event including PTEN deletion, AKT3 amplification and aberrant growth factors signaling. Recently, we showed that autocrine production of SPARC by melanoma cells participates to elevated levels of AKT activation and increased tumor cell survival [36]. Besides the involvement of AKT in survival, growth and metabolic-related pathways, AKT affects cell motility and invasion, prerequisite processes for metastasis [45], [46]. Interestingly, AKT was shown to downregulate E-cadherin expression and to promote EMT-like transition and invasiveness in carcinoma cells by inducing SNAIL [47]. Our study showing that AKT also contributes to SLUG regulation downstream SPARC-induced EMT-like changes in melanomas emphasizes the crucial role of AKT in controlling SNAIL family factors and tumor-associated EMT processes. However, the mechanisms by which AKT activates SLUG expression and transcriptional activity remain unclear and need further investigations.

We recently described an alternate role for SPARC in melanoma progression, namely its suppression of p53-dependent responses. Inactivation of p53 by SPARC resulted from AKT-dependent activation of MDM2 and we demonstrated that p53 is activated upon knockdown of SPARC in melanoma cells [36]. Recently, p53 was shown to promote SLUG degradation in lung carcinoma cells [37]. We therefore examined the regulation of SLUG by p53 in SPARC knockdown cells and found that SLUG regulation by SPARC is independent of both expression and mutational status of p53. Accordingly, we observed that reversion of the EMT-like process, including E- and P-cadherin derepression, did not require activation of p53. Consistently, we also reported that decreased motility and invasion of SPARC deficient cells is independent of p53 genetic status [25]. These observations support the notion that SPARC controls two AKT-dependent pathways involved in (i) melanoma cell survival through p53; and (ii) EMT-associated cell migration through SLUG. Interestingly, our analysis of actin cytoskeleton revealed that suppression or overexpression of SPARC results in a similar phenotype i.e. an increase of stress fibers formation. The molecular mechanisms that are involved remain unknown, but this observation outlines the role of SPARC in actin network plasticity.

SLUG is a potent inducer of cell movement during development [48], epithelial cell migration [14] and tumor cell motility [1]. Here we found that SLUG knockdown cytoskeletal organization, abrogates melanoma cell migration and tumor invasiveness, further supporting the role of SLUG in tumor motility. However, it remains still unclear how SLUG controls these processes. It has been reported that SLUG controls integrin expression in human epidermal keratinocytes [49]. Integrin-dependent cell-matrix substratum interaction is essential for directing cell movement. Interestingly, our findings show that several integrin-dependent events are altered in SLUG knockdown cells, such as adhesion on Fibronectin, tumor invasiveness in 3D collagen matrix, cell morphology and actin cytoskeleton, suggesting that abnormal cell migration observed in SLUG silenced cells result from impaired integrin signaling and adhesion. Whether this results from a direct transcriptional repression of integrin expression or indirect changes in integrin inside-out signaling is currently under investigation in our laboratory.

SLUG knockdown increases Ca^2+^-dependent cell-cell adhesion and, in addition to E-cadherin, our data demonstrate that SLUG regulates P-cadherin expression in melanoma cells. P-cadherin was described as a regulator of cell-cell adhesion and melanoma invasiveness [50]. Down-regulation of P-cadherin is a frequent event observed during melanoma progression [7]. However, the regulatory mechanism of P-cadherin suppression is unknown. To our knowledge, our findings that SLUG controls P-cadherin levels in melanomas cells are the first description of such a mechanism. In addition, we provide here evidence that SPARC constitutes an important upstream regulator of P-cadherin in melanocytes and melanoma cells. However, it remains to be shown whether P-cadherin is a transcriptional target of SLUG in EMT-associated events.

In melanoma, SLUG functions as a melanocyte-specific factor required for the promotion of the metastatic phenotype [15]. The signaling pathways controlling SLUG expression in melanoma cells are beginning to be unraveled. Recently, SLUG was reported to be regulated by HGF-dependent signaling pathways in human melanoma cells [51], and by TGF-beta and activin A in B16 murine melanoma cells [52]. Here, we show that SLUG is controlled by the PI3 kinase/AKT signaling pathway downstream of the matricellular SPARC protein, connecting for the first time SLUG levels to extracellular environment cues critical for the acquisition of the invasive phenotype.

The transcriptional factors of the SNAIL family SNAIL and SLUG were recently shown to collaborate on local tumor invasion and promotion of distant metastasis [53]. In addition, a sequential, hierarchical action of SNAIL factors during tumor progression has been proposed. However, it was reported that SNAIL and SLUG play distinct roles in breast carcinoma progression [54]. Thus, it would be of great importance to investigate whether SNAIL and SLUG cooperate or act independently in the acquisition of invasiveness downstream of SPARC. We previously observed an inversed correlation between SPARC and E-cadherin levels in melanocytes and melanoma cells [22]. Interestingly, in some melanomas SNAIL and SPARC levels were not correlated, suggesting that mechanisms other than SNAIL induction may be involved in SPARC-mediated E-cadherin repression. Although the melanoma sample size analyzed here is relatively small, the results indicate that SPARC and SLUG expression is positively associated during melanoma progression, further underlining the importance of our present observations. Additional analyses on a larger clinical collection of the expression of SPARC, SNAIL, SLUG and E-cadherin are clearly warranted.

In conclusion, we report here the first demonstration of a regulation of the lineage-specific developmental factor SLUG by SPARC through an AKT-dependent pathway and the importance of this mechanism in EMT-induced cell invasion in melanoma.

## Supporting Information

Figure S1 **SPARC induces mesenchymal-like transition.** (**A**) **Effect of SPARC overexpression on expression of SLUG, SNAIL, TBX2 and TBX3 in 501mel cells.** Immunoblots of 501mel cells infected with control adenovirus (AdCTRL) or adenovirus-expressing SPARC (AdSPARC). Total protein lysates were analyzed for expression of SPARC-Myc transgene, endogenous SPARC, SLUG, SNAIL, TBX2 and TBX3. HSP60 was used as loading control. (**B**) **Immunoblot analyses showing the increase of Fibronectin in normal human melanocytes cells infected by AdSPARC.** Densitometric analysis of three independent Western blots for Fibronectin is shown. *P<0.05 (Student’s test). (**C**) **SPARC overexpression increases SLUG mRNA levels.** RNAs were prepared from 501mel cells overexpressing SPARC (cl. SPARC #1 and #2) or control vector (cl. CTRL). Slug mRNA expression was measured by SYBR green-based real-time Q-PCR. Relative expression level of SLUG mRNA was normalized for RNA concentrations with four different housekeeping genes. *Columns*, mean of two independent amplifications performed in duplicate; *error bars*, SD. *P<0.05 (Student’s test). (**D**) **Viability of melanoma cells after 4 days of SPARC knockdown by siRNA.** The indicated melanoma cells were transfected with control siRNA (siCTRL, open bars) or SPARC siRNA (siSPARC, filled bars) at 50 nM. 4 days after transfection, cell proliferation was measured by XTT assay. Results are expressed in percent of control. *Columns*, mean of 4 independent determinations; *error bars*, SD. *NS*, not significant (Student’s test).
(TIF)

Figure S2 **siRNA-mediated SLUG knockdown restores E-cadherin expression through transcriptional derepression of the promoter.** (**A**) **Analysis of E- and P-cadherin protein levels in SLUG-depleted cells.** The indicated melanoma cells were transfected with control siRNA (siCTRL) for 4 days or SLUG siRNA (siSLUG) for the indicated times. Expression levels of SLUG, E-cadherin and P-cadherin were analyzed by immunoblotting. HSP60 was used as loading control. (**B**) **E-cadherin mRNA levels following SLUG or SPARC depletion.** RNAs were prepared from WM9 cells transfected with siCTRL, siSPARC or siSLUG for 3 days. The relative mRNA expression levels of SPARC, SLUG and E-cadherin were measured by SYBR green-based real-time Q-PCR. *P<0.05 (Student’s test). (**C**) **E-cadherin promoter activity following SLUG or SPARC depletion.** Cells were transfected with siCTRL, siSPARC or siSLUG, and 24 hours later with wild-type E-cadherin promoter reporter construct. After 3 days, luciferase activities were measured and normalized to β-galactosidase activities. *Columns*, mean of triplicates; *errors bars*, SD. (**D**) **E-cadherin promoter activity following SLUG or SPARC expression.** Cells were co-transfected with an empty vector (mock) or vectors expressing SPARC or SLUG, and wild-type E-cadherin promoter reporter construct as indicated. Measurement of luciferase activities was assessed as described above.
(TIF)

Figure S3 **siRNA-mediated SPARC or SLUG knockdown impairs melanoma cell migration.** 501mel (**A**), WM9 (**B**), MeWo (**C**) and SKmel28 (**D**) cells were transfected with control siRNA (siCTRL), two SPARC siRNAs (siSPARC #1 and #2) or two SLUG siRNAs (siSLUG #1 and #2) for 4 days. Serum-stimulated cell migration was assessed using Boyden chamber assays. Cells were left to migrate for 20 hours, then fixed, stained and counted. Results are expressed in percent of control. *Columns*, means of triplicates from two independent experiments; *error bars*, SD. Representative images of lower surface of membranes are shown.
(TIF)

Figure S4 **siRNA-mediated SLUG knockdown shows no effect on melanoma cell proliferation, cell cycle progression or apoptosis.** (**A**) **Analysis of cell proliferation.** The indicated melanoma cells were transfected with control siRNA (siCTRL, open bars) or SLUG siRNA (siSLUG, filled bars) at 50 nM. 4 days after transfection, cell proliferation was measured by XTT assay (**B**) **Analysis of cell cycle distribution.** The indicated melanoma cells were transfected with siCTRL or siSLUG, stained with PI and analyzed for DNA content by flow cytometry. Histograms represent the percentage of cells in different phases of the cell cycle. (**C**) **Analysis of PARP cleavage.** 501mel cells were transfected with control siRNA (siCTRL) or SLUG siRNA (siSLUG) for 4 days. Expression levels of SLUG and cleaved PARP were analyzed by immunoblotting. Treatment with Staurosporine for 10 hours was used as a positive control of apoptotic cell death. HSP60 was used as loading control (**D**) **Analysis of cell apoptosis.** WM9 and SKmel 28 cells were transfected with control siRNA (siCTRL) or SLUG siRNA (siSLUG) for 4 days or treated with Staurosporine as above. Cells were stained with PI and Annexin-V-fluos and analyzed by flow cytometry. Histograms show Annexin-V positive/PI negative cells (filled bars; apoptotic subpopulation) and both Annexin-V/PI positive cells (open bars; post-apoptotic/necrotic subpopulation). Note that unlike Staurosporine treatment, cells depleted for SLUG did not undergo apoptosis.
(TIF)

Figure S5 **siRNA-mediated SLUG knockdown decreases adhesion to Fibronectin.** (**A**) **Time-course of adhesion to Fibronectin.** 501mel cells were transfected with control siRNA (siCTRL) or SLUG siRNA (siSLUG) for 4 days, then detached, loaded with the green fluorescent marker CMFDA and plated on Fibronectin-coated wells. At the indicated time points, cells were washed and fluorescence of adherent cells was determined with a microplate reader. (**B**) **Adhesion assays in SLUG-depleted MeWo and SKmel28 cells.** Fluorescent adhesion assays on Fibronectin were performed after transfection of MeWo and SKmel28 cells with control siRNA (siCTRL) or SLUG siRNA (siSLUG) for 4 days. Cells were left to adhere for 3 hours and analyzed as above. *Columns*, average of two independent adhesion assays; *error bars*, SD. *P<0.05 (Student’s test).
(TIF)
