# Supplementary figures and images for: The Epithelial-Mesenchymal Transition (EMT) Regulatory Factor SLUG (SNAI2) Is a Downstream Target of SPARC and AKT in Promoting Melanoma Cell Invasion
Source: PLoS One. 2012 Jul 20;7(7):e40378. doi: 10.1371/journal.pone.0040378 (PMC3401237; doi:10.1371/journal.pone.0040378)

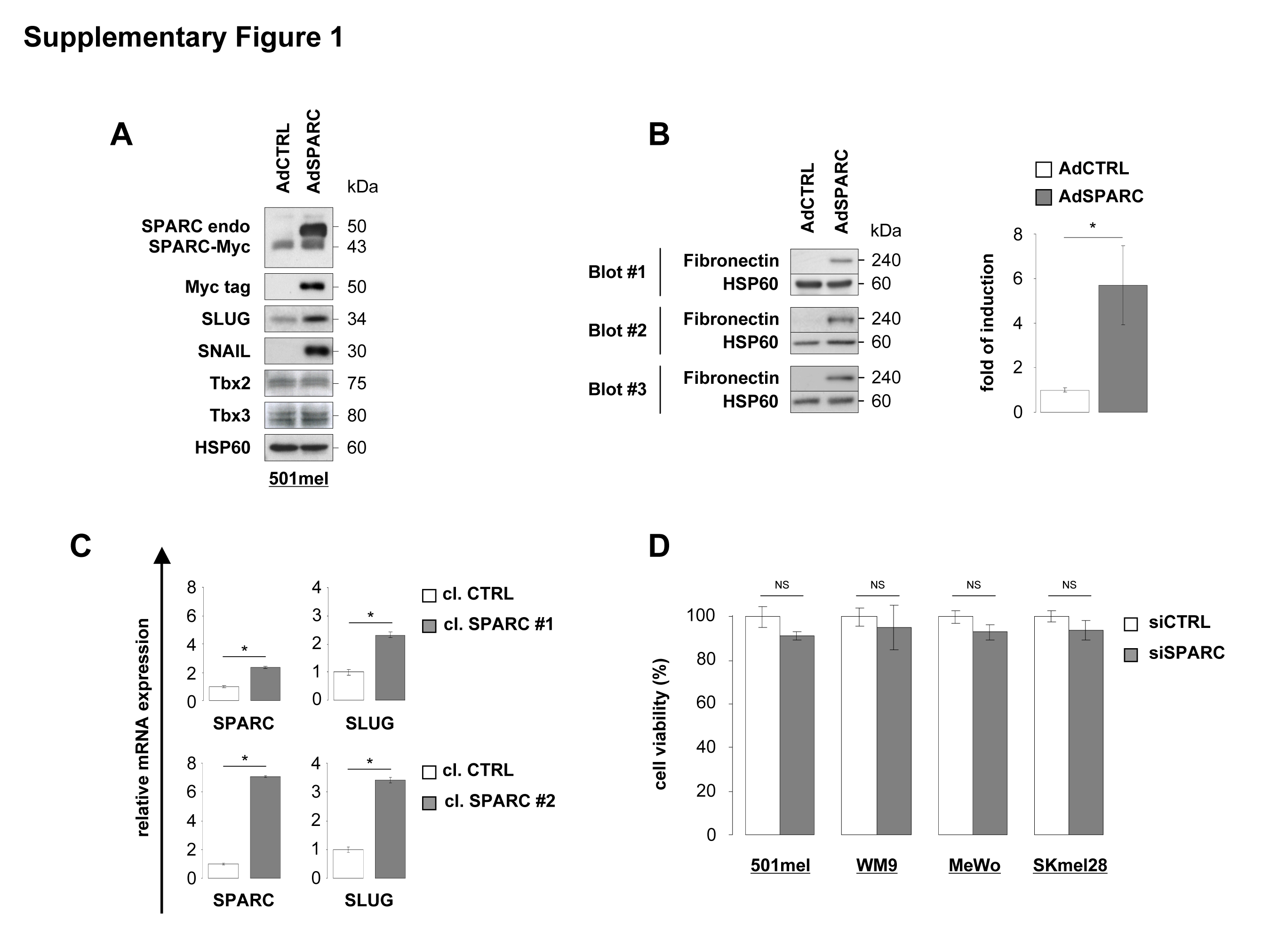

Supplement: Figure S1 — SPARC induces mesenchymal-like transition. (A) Effect of SPARC overexpression on expression of SLUG, SNAIL, TBX2 and TBX3 in 501mel cells. Immunoblots of 501mel cells infected with control adenovirus (AdCTRL) or adenovirus-expressing SPARC (AdSPARC). Total protein lysates were analyzed for expression of SPARC-Myc transgene, endogenous SPARC, SLUG, SNAIL, TBX2 and TBX3. HSP60 was used as loading control. (B) Immunoblot analyses showing the increase of Fibronectin in normal human melanocytes cells infected by AdSPARC. Densitometric analysis of three independent Western blots for Fibronectin is shown. *P<0.05 (Student’s test). (C) SPARC overexpression increases SLUG mRNA levels. RNAs were prepared from 501mel cells overexpressing SPARC (cl. SPARC #1 and #2) or control vector (cl. CTRL). Slug mRNA expression was measured by SYBR green-based real-time Q-PCR. Relative expression level of SLUG mRNA was normalized for RNA concentrations with four different housekeeping genes. Columns, mean of two independent amplifications performed in duplicate; error bars, SD. *P<0.05 (Student’s test). (D) Viability of melanoma cells after 4 days of SPARC knockdown by siRNA. The indicated melanoma cells were transfected with control siRNA (siCTRL, open bars) or SPARC siRNA (siSPARC, filled bars) at 50 nM. 4 days after transfection, cell proliferation was measured by XTT assay. Results are expressed in percent of control. Columns, mean of 4 independent determinations; error bars, SD. NS, not significant (Student’s test). (TIF) [file pone.0040378.s001.tif]

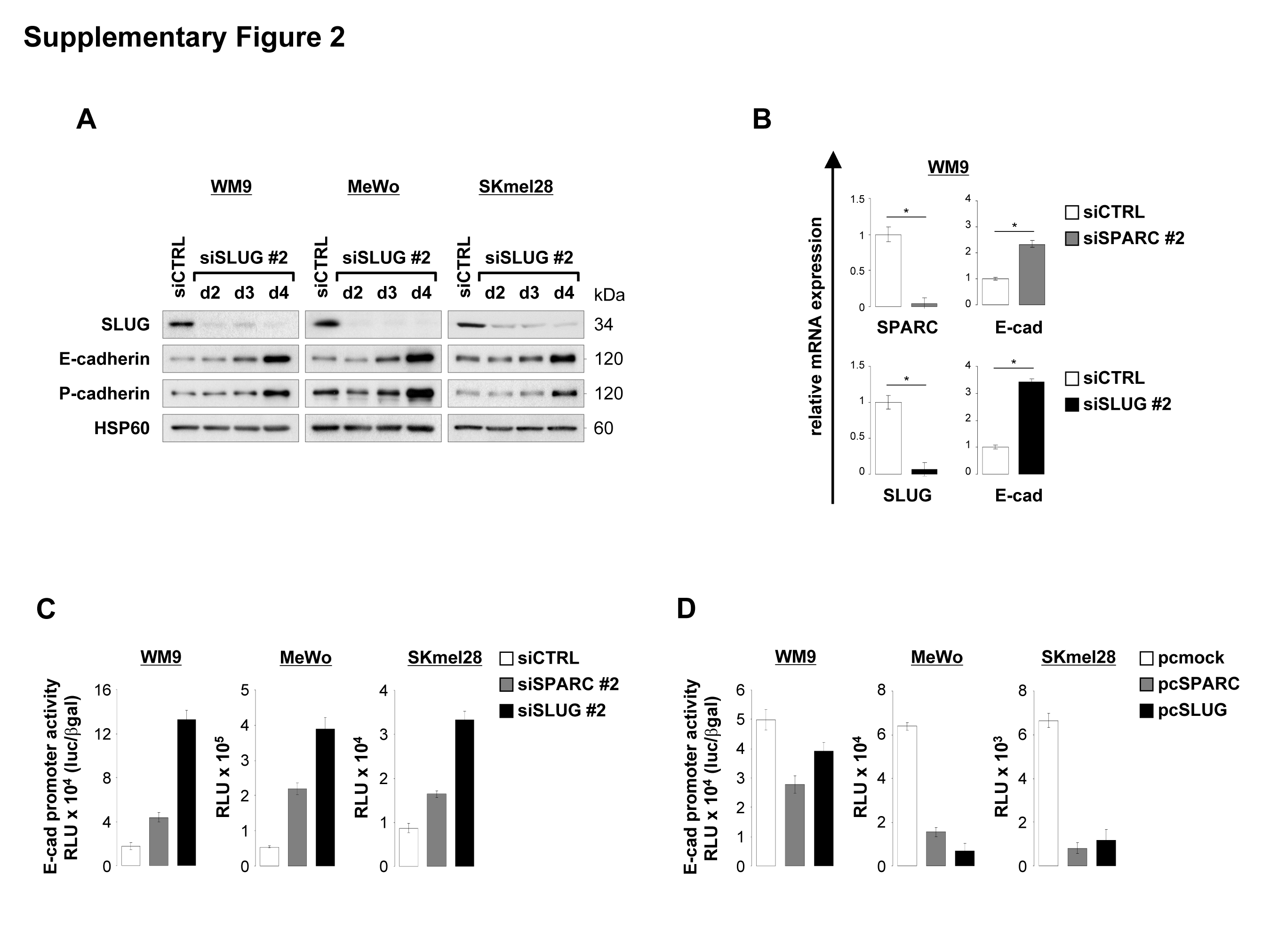

Supplement: Figure S2 — siRNA-mediated SLUG knockdown restores E-cadherin expression through transcriptional derepression of the promoter. (A) Analysis of E- and P-cadherin protein levels in SLUG-depleted cells. The indicated melanoma cells were transfected with control siRNA (siCTRL) for 4 days or SLUG siRNA (siSLUG) for the indicated times. Expression levels of SLUG, E-cadherin and P-cadherin were analyzed by immunoblotting. HSP60 was used as loading control. (B) E-cadherin mRNA levels following SLUG or SPARC depletion. RNAs were prepared from WM9 cells transfected with siCTRL, siSPARC or siSLUG for 3 days. The relative mRNA expression levels of SPARC, SLUG and E-cadherin were measured by SYBR green-based real-time Q-PCR. *P<0.05 (Student’s test). (C) E-cadherin promoter activity following SLUG or SPARC depletion. Cells were transfected with siCTRL, siSPARC or siSLUG, and 24 hours later with wild-type E-cadherin promoter reporter construct. After 3 days, luciferase activities were measured and normalized to β-galactosidase activities. Columns, mean of triplicates; errors bars, SD. (D) E-cadherin promoter activity following SLUG or SPARC expression. Cells were co-transfected with an empty vector (mock) or vectors expressing SPARC or SLUG, and wild-type E-cadherin promoter reporter construct as indicated. Measurement of luciferase activities was assessed as described above. (TIF) [file pone.0040378.s002.tif]

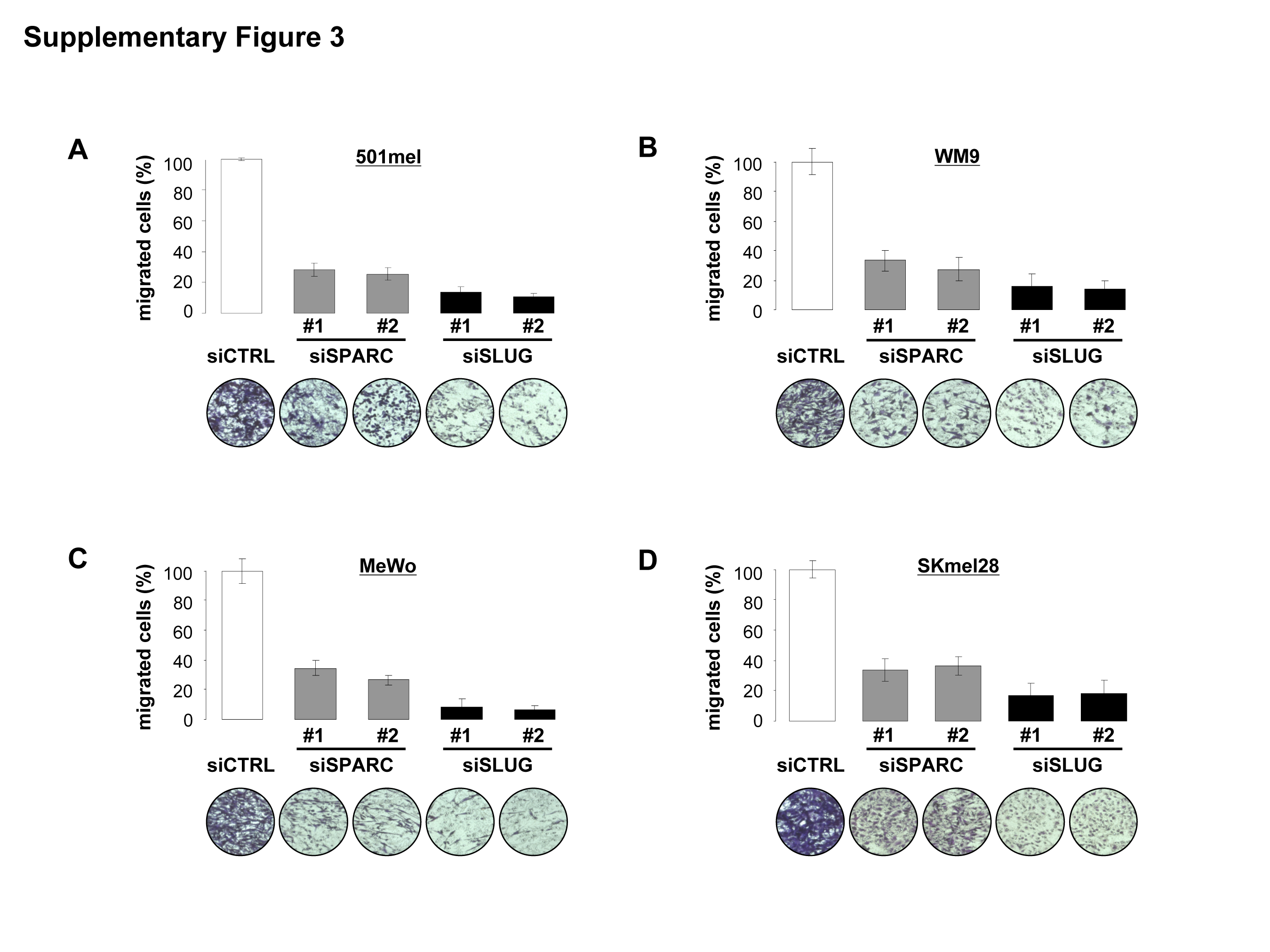

Supplement: Figure S3 — siRNA-mediated SPARC or SLUG knockdown impairs melanoma cell migration. 501mel (A), WM9 (B), MeWo (C) and SKmel28 (D) cells were transfected with control siRNA (siCTRL), two SPARC siRNAs (siSPARC #1 and #2) or two SLUG siRNAs (siSLUG #1 and #2) for 4 days. Serum-stimulated cell migration was assessed using Boyden chamber assays. Cells were left to migrate for 20 hours, then fixed, stained and counted. Results are expressed in percent of control. Columns, means of triplicates from two independent experiments; error bars, SD. Representative images of lower surface of membranes are shown. (TIF) [file pone.0040378.s003.tif]

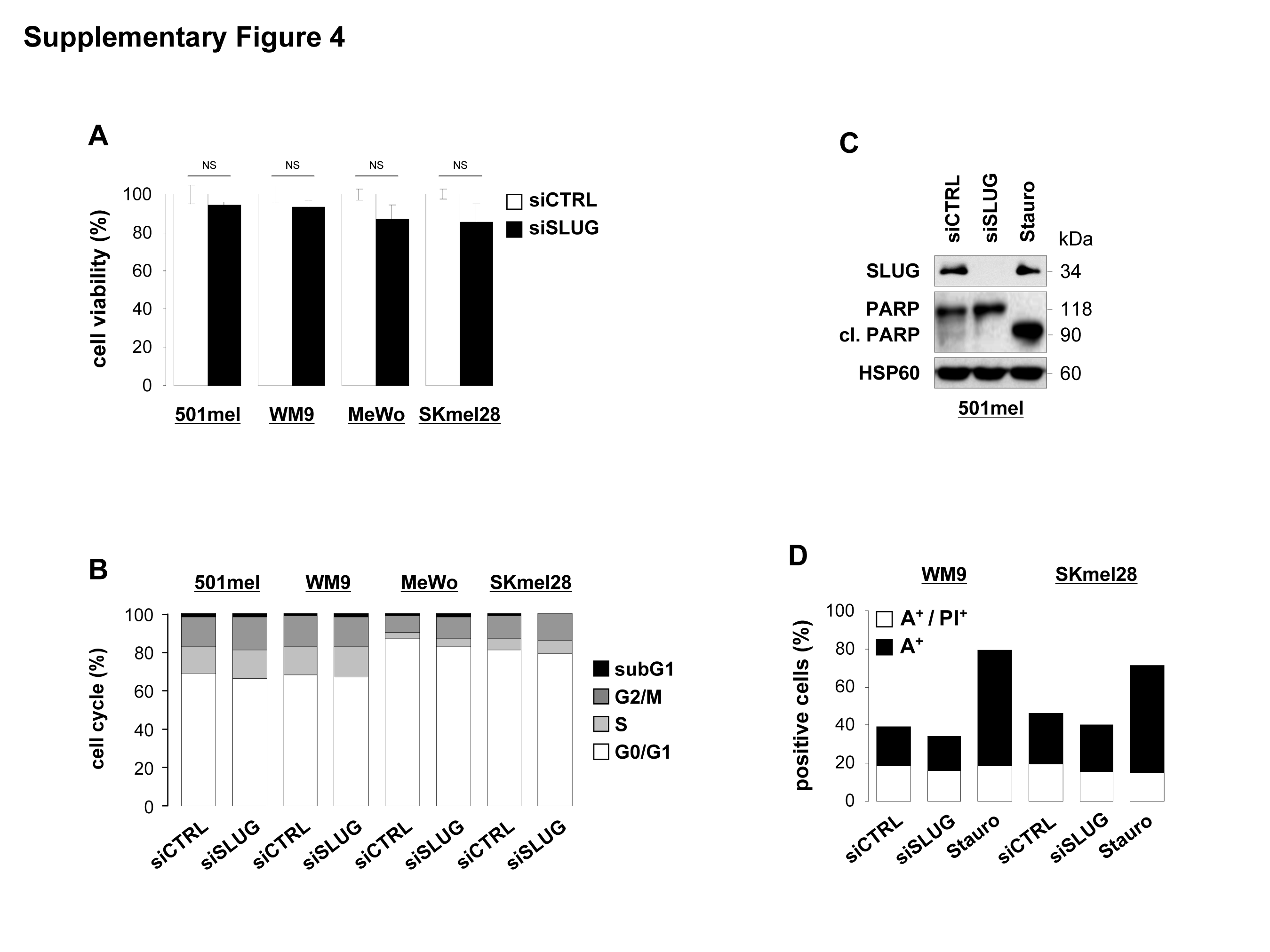

Supplement: Figure S4 — siRNA-mediated SLUG knockdown shows no effect on melanoma cell proliferation, cell cycle progression or apoptosis. (A) Analysis of cell proliferation. The indicated melanoma cells were transfected with control siRNA (siCTRL, open bars) or SLUG siRNA (siSLUG, filled bars) at 50 nM. 4 days after transfection, cell proliferation was measured by XTT assay (B) Analysis of cell cycle distribution. The indicated melanoma cells were transfected with siCTRL or siSLUG, stained with PI and analyzed for DNA content by flow cytometry. Histograms represent the percentage of cells in different phases of the cell cycle. (C) Analysis of PARP cleavage. 501mel cells were transfected with control siRNA (siCTRL) or SLUG siRNA (siSLUG) for 4 days. Expression levels of SLUG and cleaved PARP were analyzed by immunoblotting. Treatment with Staurosporine for 10 hours was used as a positive control of apoptotic cell death. HSP60 was used as loading control (D) Analysis of cell apoptosis. WM9 and SKmel 28 cells were transfected with control siRNA (siCTRL) or SLUG siRNA (siSLUG) for 4 days or treated with Staurosporine as above. Cells were stained with PI and Annexin-V-fluos and analyzed by flow cytometry. Histograms show Annexin-V positive/PI negative cells (filled bars; apoptotic subpopulation) and both Annexin-V/PI positive cells (open bars; post-apoptotic/necrotic subpopulation). Note that unlike Staurosporine treatment, cells depleted for SLUG did not undergo apoptosis. (TIF) [file pone.0040378.s004.tif]

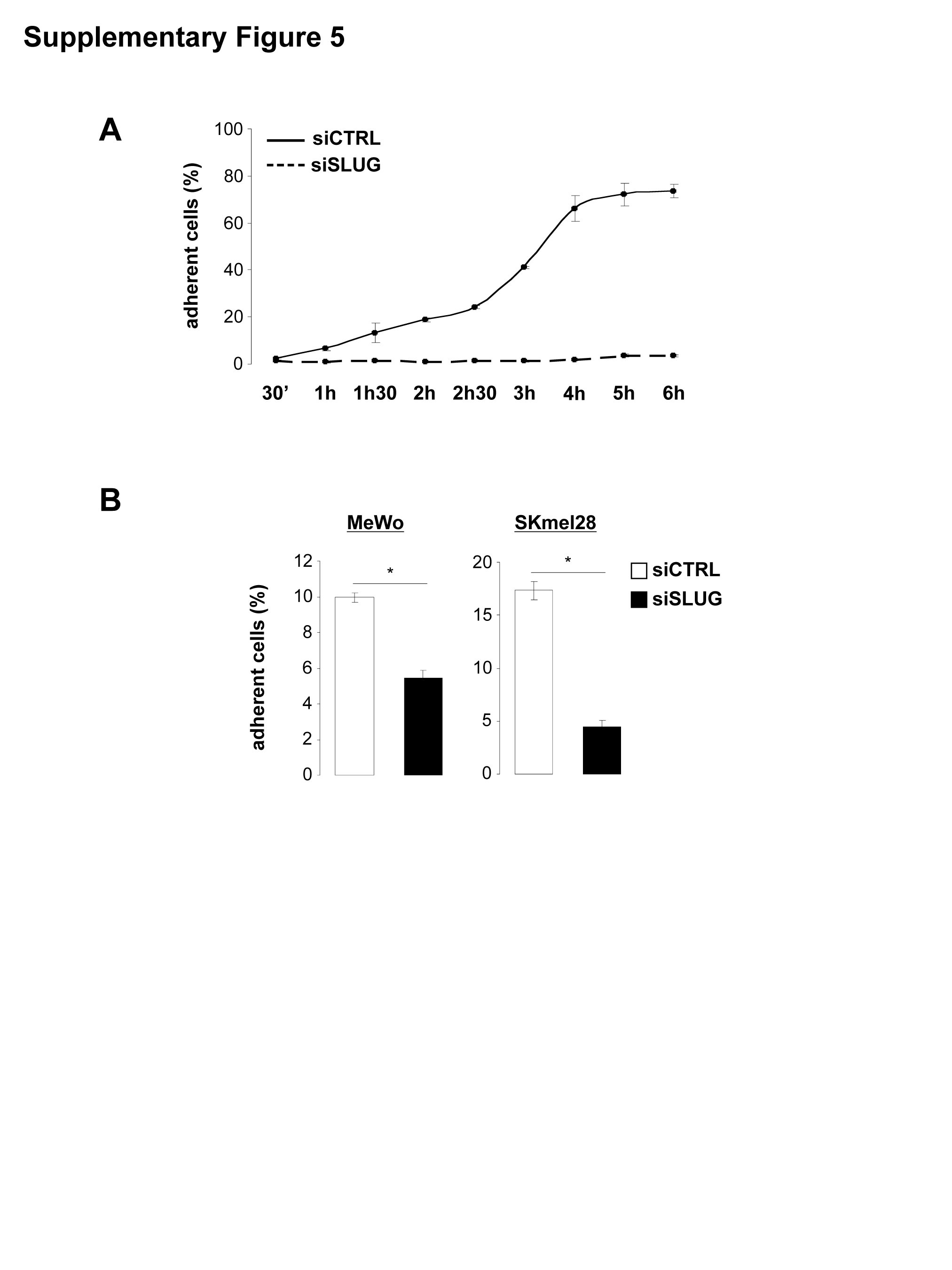

Supplement: Figure S5 — siRNA-mediated SLUG knockdown decreases adhesion to Fibronectin. (A) Time-course of adhesion to Fibronectin. 501mel cells were transfected with control siRNA (siCTRL) or SLUG siRNA (siSLUG) for 4 days, then detached, loaded with the green fluorescent marker CMFDA and plated on Fibronectin-coated wells. At the indicated time points, cells were washed and fluorescence of adherent cells was determined with a microplate reader. (B) Adhesion assays in SLUG-depleted MeWo and SKmel28 cells. Fluorescent adhesion assays on Fibronectin were performed after transfection of MeWo and SKmel28 cells with control siRNA (siCTRL) or SLUG siRNA (siSLUG) for 4 days. Cells were left to adhere for 3 hours and analyzed as above. Columns, average of two independent adhesion assays; error bars, SD. *P<0.05 (Student’s test). (TIF) [file pone.0040378.s005.tif]
